# I-DWRL: Improved Dual Wireless Radio Localization Using Magnetometer

**DOI:** 10.3390/s17112630

**Published:** 2017-11-15

**Authors:** Abdul Aziz, Ramesh Kumar, Inwhee Joe

**Affiliations:** 1 Department of Electronics and Computer Engineering, Hanyang University, Seoul 04763, Korea; azizsheraz@yahoo.com; 2 Department of Electronic Engineering, Dawood University of Engineering and Technology, Karachi 74800, Pakistan; rameshkarmani@gmail.com; 3 Division of Computer Science and Engineering, Hanyang University, Seoul 04763, Korea

**Keywords:** DWRL, I-DWRL, UWB, dual radio, magnetometer, localization, wireless sensor network

## Abstract

In the dual wireless radio localization (DWRL) technique each sensor node is equipped with two ultra-wide band (UWB) radios; the distance between the two radios is a few tens of centimeters. For localization, the DWRL technique must use at least two pre-localized nodes to fully localize an unlocalized node. Moreover, in the DWRL technique it is also not possible for two sensor nodes to properly communicate location information unless each of the four UWB radios of two communicating sensor nodes cannot approach the remaining three radios. In this paper, we propose an improved DWRL (I-DWRL) algorithm along with mounting a magnetometer sensor on one of the UWB radios of all sensor nodes. This addition of a magnetometer helps to improve DWRL algorithm such that only one localized sensor node is required for the localization of an unlocalized sensor node, and localization can also be achieved even when some of the four radios of two nodes are unable to communicate with the remaining three radios. The results show that with the use of a magnetometer a greater number of nodes can be localized with a smaller transmission range, less energy and a shorter period of time. In comparison with the conventional DWRL algorithm, our I-DWRL not only maintains the localization error but also requires around half of semi-localizations, 60% of the time, 70% of the energy and a shorter communication range to fully localize an entire network. Moreover, I-DWRL can even localize more nodes while transmission range is not sufficient for DWRL algorithm.

## 1. Introduction

Wireless sensor networks have been a vital research area for many years. Nowadays sensor nodes are used widely in variety of applications, devices, buildings, vehicles, and so forth. The first research in this area was motivated by military applications, with defense advanced research projects agency (DARPA) funding a number of prominent research projects such as Smart Dust, and neural engineering science and technology (NEST) [1].

Wireless sensor networks (WSNs) consist of collection of small, low-cost, usually randomly placed heterogeneous sensor nodes connected by wireless media to form a sensor field. Nodes monitor the environment, gather data and send it to the sink node through single or multichip communications. Its many applications include battlefield surveillance, habitat monitoring, environmental monitoring, health applications, target tracking, event detection, vehicle tracking, and forest fire detection [2].

To make the collected information valuable, many applications such as geographical routing and location-based information applications require the exact locations of deployed sensor nodes. Therefore, many location-finding schemes have been proposed for wireless sensor networks.

This paper is organized as follows. Section 2 covers related work and details of the dual wireless radio localization (DWRL) algorithm. An improved dual wireless radio localization (I-DWRL) scheme is presented in Section 3. Section 4 presents the simulation work and results, and then we conclude the paper in Section 5.

## 2. Related Work

Localization in WSN is an active area of research with several surveys [3,4,5,6,7,8,9,10,11] and more than 50 localization algorithms. Localization techniques can be generally categorized as the following:

- absolute vs. relative
- centralized vs. decentralized
- range free vs. range based
- anchor vs. anchorless.

Absolute localization is to locate sensor nodes with respect to coordinate system, whereas in relative localization, the location of sensor nodes is found in relation to other sensors. Global positioning system is an example of absolute localization [12].

In centralized localization [13,14], one central base station is present for computation. The disadvantage is the overhead, and the cost also increases. Multidimensional scaling map (MDS-MAP) [14] is a centralized algorithm for computing the coordinates of unknown nodes after approximating the distances between the nodes using the shortest path algorithm. In contrast, decentralized or distributed localization techniques [15,16] depend on each sensor node being able to determine its location with only limited communication with nearby nodes.

Range-free [17] techniques depend upon factors such as the number of hop counts and connectivity, whereas range-based techniques include received signal strength indication (RSSI) [18], time of arrival (ToA) [19], angle of arrival (AoA) [20], time difference of arrival (TDoA), lateration and angulation, and so forth [10]. There is wide range of radio signals but ultrawide band (UWB) signals are particularly well suited for range-based localization, since they can provide accurate and reliable range measurements due to their fine delay resolution and robustness in harsh environments [21,22,23,24].

The anchor-based algorithms provide a starting point for an algorithm by using the position of anchor nodes. In contrast, anchorless schemes measure the distance between nodes for creating a local map of the nodes. The local map created is not a unique one and can be stitched to any coordinate system with the help of translation, rotation or flipping.

DWRL and I-DWRL algorithms fall into the category of range-based localization where an anchor node is also used to start the localization process. Both algorithms are designed for coplanar static wireless sensor and ad hoc networks.

### 2.1. Dual Wireless Radio Localization

**Figure 1:**
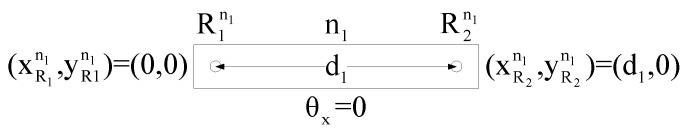
Dual wireless radio node.

The dual wireless radio localization (DWRL) algorithm [25] is a Global Positioning System (GPS) free-range based dual radio wireless localization algorithm for static wireless networks, where each node $n_{x}$ has two UWB wireless radios named Radio1 ($R_{1}^{n_{x}}$) and Radio2 ($R_{2}^{n_{x}}$) that are attached to an a priori known positions on board, as shown in Figure 1. The straight line joining $R_{1}^{n_{x}}$ and $R_{2}^{n_{x}}$ is considered as the axis of node. In this technique one of the nodes is designated as sink node whose $R_{1}^{n_{x}}$ is set at origin ${(x}_{R_{1}}^{n_{x}}{,y}_{R_{1}}^{n_{x}})\;=\;(0,0)$ and $R_{2}^{n_{x}}$ in the direction of positive *x*-axis ${(x}_{R_{2}}^{n_{x}}{,y}_{R_{2}}^{n_{x}}{)\;=\;(d}_{x},0)$, which means the angle $\theta_{x}$ between the x-axis and the axis of the node is zero and, here, $d_{x}$ is the distance between $R_{1}^{n_{x}}$ and $R_{2}^{n_{x}}$.

Two nodes are said to be collinear if there is a straight line passing through all four radios of the nodes. The authors of the DWRL algorithm have experimentally shown in their paper and suggested that, for a typical wireless sensor node, the inter-radio distance of 60 cm is sufficient for successful localization [25]. While an increased inter-radio distance improves localization accuracy, in all of our simulation scenarios we consider the minimum inter-radio distance of 60 cm to avoid a greater size of sensor nodes for practical scenarios.

DWRL algorithm is presented in three steps, which are presented below.

#### 2.1.1. Semi-Localization

**Figure 2:**
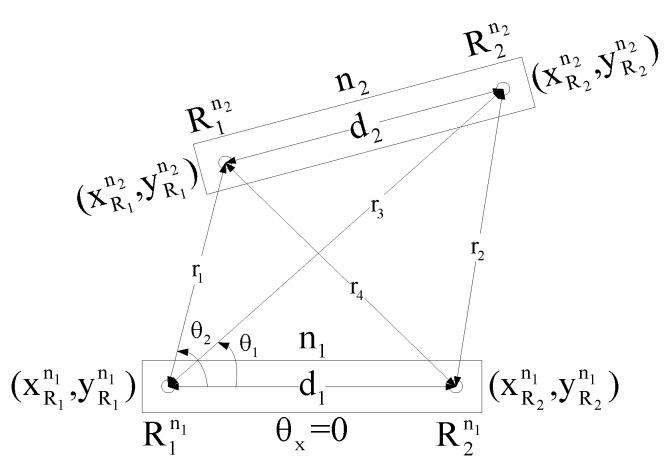
Semi Localization between node $n_{1}$ and $n_{2}$.

The first step towards the localization of any unlocalized node with the help of a sink node or some other already localized node is semi-localization. For localization only, UWB radio ranging is used to measure four distances—$r_{1}$, $r_{2}$, $r_{3}$ and $r_{4}$—between the radios of the two nodes that are within range of each other, as shown in Figure 2. Here $n_{1}$ is the sink node, and $n_{2}$ is an unlocalized node, where $d_{1}$ and $d_{2}$ are already known radio separation distances. By using the law of cosines as given in Equations (1)–(8), two solutions are obtained for the location of node $n_{2}$. Figure 2 shows semi-localization between node $n_{1}$ and $n_{2}$.

(1) $$r_{2}^{2}{=\;d}_{1}^{2}{+r}_{3}^{2}-{2d}_{1}r_{3}{\cos\theta }_{1}$$

(2) $$r_{4}^{2}{=\;d}_{1}^{2}{+r}_{1}^{2}-{2d}_{1}r_{1}{\cos\theta }_{2}$$

(3) $$\theta_{1}=\pm \cos^{-1}(\frac{d_{1}^{2}{+r}_{3}^{2}-r_{2}^{2}}{{2d}_{1}r_{3}})$$

(4) $$\theta_{2}=\pm \cos^{-1}(\frac{d_{1}^{2}{+r}_{1}^{2}-r_{4}^{2}}{{2d}_{1}r_{1}})$$

(5) $$x_{R_{1}}^{n_{2}}{\;=\;r}_{1}{\cos\theta }_{2}$$

(6) $$y_{R_{1}}^{n_{2}}=\pm r_{1}{\sin\theta }_{2}$$

(7) $$x_{R_{2}}^{n_{2}}{=\;r}_{3}{\cos\theta }_{1}$$

(8) $$y_{R_{2}}^{n_{2}}=\pm r_{3}{\sin\theta }_{1}$$

**Figure 3:**
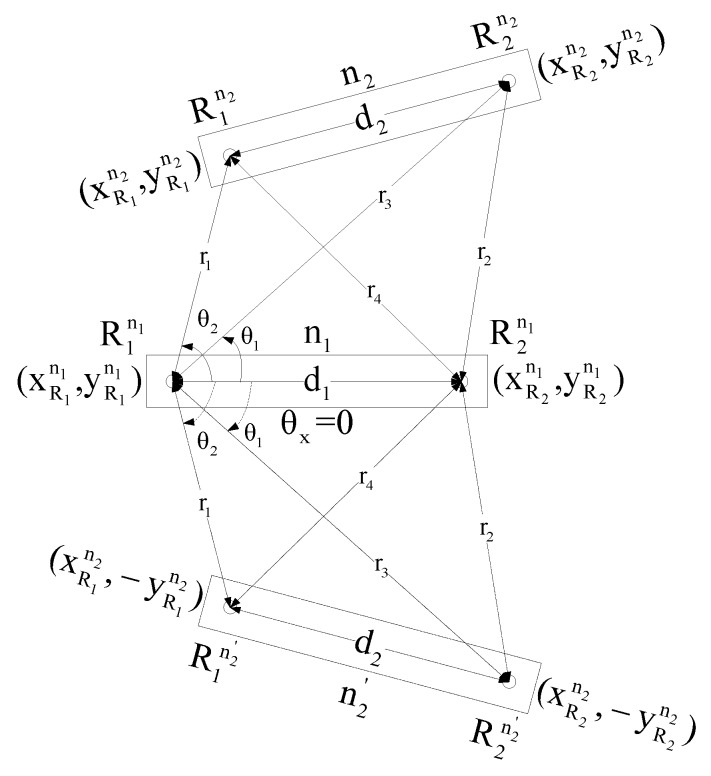
Two possible positions of node $n_{2}$ (i.e., $n_{2}$ and $n_{2}^{\prime }$).

Figure 3 shows two solutions for the position of node $n_{2}$: one is the actual position and the other position is located symmetrically on a flip around the axis of node $n_{1}$. There will be only one solution if and only if the axes of $n_{1}$ and $n_{2}$ lie on a same straight line or, in other words, both nodes are collinear.

#### 2.1.2. Rigid Localization

**Figure 4:**
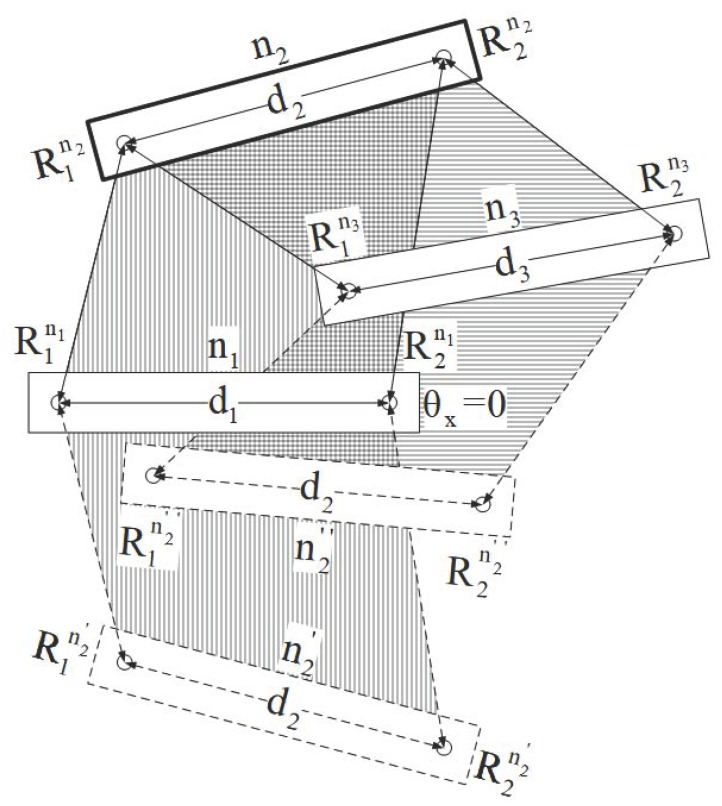
Rigid localization of node $n_{2}$ using localized nodes $n_{1}$ and $n_{3}$.

As in semi-localization, if the two nodes $n_{1}$ and $n_{2}$ are collinear, then both the location solutions will be same, and will indicate actual position of node $n_{2}$. If both the nodes are not collinear then there is a need of the rigid localization. Rigid localization makes use of an additional node with additional semi-localization for selecting the correct solution for node $n_{2}$ by matching the location that is the best overlapped from the $n_{1}$ − $n_{2}$ and $n_{3}$ − $n_{2}$ semi-localization steps, where nodes $n_{1}$ and $n_{3}$ are already localized. In Figure 4 node $n_{2}$ is rigid localized with the help of node $n_{1}$ and $n_{3}$, which are somehow already rigid localized. Here, $n_{2}^{\prime }$ and $n_{2}^{\prime \prime }$ are not the actual positions of $n_{2}$.

#### 2.1.3. DWRL Algorithm

**Figure 5:**
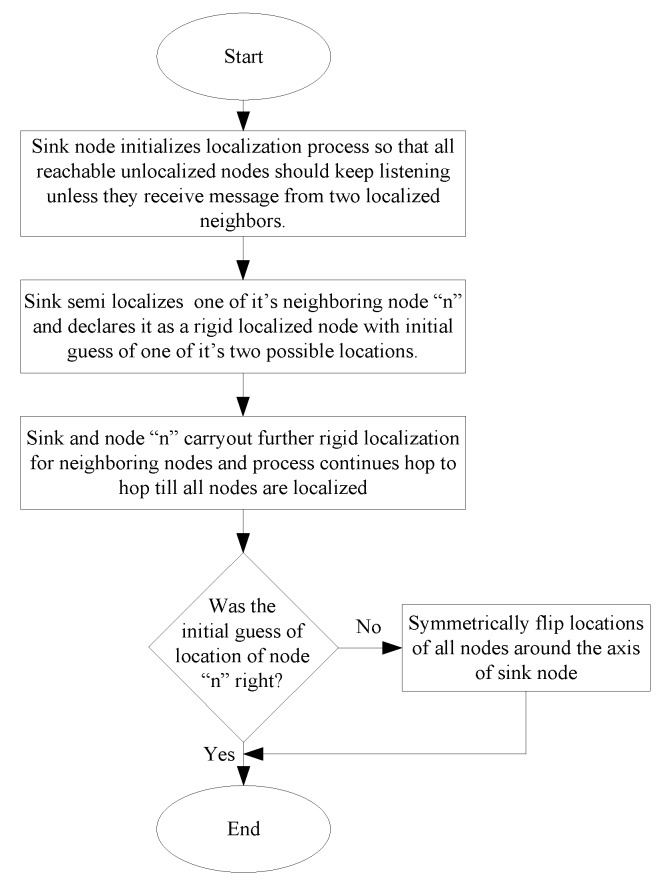
Flowchart of dual wireless radio localization (DWRL) algorithm.

The sink node initializes the localization process. Firstly, one of the neighbor nodes is semi-localized and declared to be rigid localized even without performing rigid localization, because the sink is the only localized node and there is no other localized node to perform rigid localization. The initial guess of the actual position of the first node is left to be verified by a third party outside of the network such as network operator, and it should be verified at the end when the whole network is localized. Later, the sink and the first localized node will rigid localize their neighbor nodes and the process continues. During the localization process, all unlocalized nodes keep listening unless they receive message from two localized neighboring nodes. Finally, a third party should check whether the initial guess of the location of the first node was not correct. In this case, the locations of all nodes should be symmetrically flipped around the axis of the sink node. Figure 5 shows flowchart of DWRL algorithm.

#### 2.1.4. DWRL Algorithm Drawbacks

Although dual radios on a single node for localization is effective, the DWRL algorithm has the following drawbacks:

- If the initial location of the sink node is not known, then the DWRL algorithm considers that Radio1 ($R_{1}^{\sin k}$) of the sink node has been assigned a specific location (0,0), and Radio2 ($R_{2}^{\sin k}$) of sink node is considered in the direction of positive x-axis, which points to local east direction. If nodes are randomly deployed, how then can we suppose the Radio2 of the sink node is in the direction of local east? The wrong angle of axis of the sink node can lead to the wrong location of the rest of the network nodes. Therefore, we need to find actual direction of $R_{2}^{\sin k}$.
- The DWRL algorithm needs at least two localized nodes to fully localize and unlocalized the node. For this reason, the DWRL algorithm cannot rigid localize first node with one sink node. First, the semi-localized node is declared as rigid localized on the basis of one randomly chosen location solution of the two possible location solutions of the first node. This can lead to serious localization problems if rest of the network continues to localize with the help of the wrong location of first node. What if the applications scenario is critical, and we have to use the location of some nodes before the whole network localization process is completed, and then the third party finds out that location of first node was not right and location of all nodes need to be flipped around the axis of the sink node? Therefore, we need to find the exact location of the first node.
- To rigid localize a node, a minimum of two semi-localizations are required. If an unlocalized node cannot listen from at least two localized nodes then it cannot be localized and it has to wait, unless somehow two localized signals are received. If some node cannot receive two localized signals then it cannot be localized at all. This requires the DWRL algorithm to perform at least (2n−3) semi-localizations [25] to localize the entire network. Here, (2n−3) is the number of semi-localizations required for network with n number of nodes, which is equal to twice the number of nodes n and three less, where three indicates that there are no semi-localizations performed for a sink node and only one semi-localization is performed for the node that will be localized first. Since (2n−3) semi-localizations can be carried out with each choice of a different edge, the total number of semi-localizations can be, at most, $P_{2}^{n}\times (2n-3)$. A higher number of semi-localizations uses more energy and time, therefore we need to minimize the total number of semi-localizations as well as increase number of fully localized nodes even with single semi-localization step.
- For successful semi-localization, each of four radios of two nodes must be in communication range with rest of the three radios. This requires either high node density or high transmission power, where high node density costs more nodes and high transmission power reduces the life of sensor nodes. We need to develop a way for nodes to be localized even if few of the four radios of the two communicating nodes can reach each other.

## 3. Improved DWRL (I-DWRL) Algorithm

**Figure 6:**
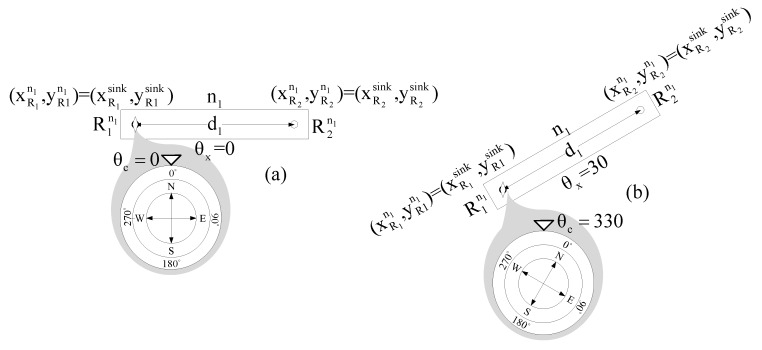
Node $n_{1}$ with Magnetometer showing different angles of magnetometer $\theta_{c}$ and $\theta_{x}$ of node in (**a**,**b**).

To present the I-DWRL algorithm, we intend to improve the drawbacks of the DWRL algorithm, as mentioned earlier. To achieve such improvements, we use a magnetometer, which is affixed on Radio1 of every single node, as shown in Figure 6. $\theta_{c}$ is the angle measured with magnetometer and ${(\theta }_{x}=360-\theta_{c})$ is the angle which roughly indicates the slope of the axis of node; it will be used in the localization process.

In I-DWRL, which is distinct from original DWRL, we have added the initial location of Radio1 of the sink node ($R_{1}^{\sin k}$) as ${(x}_{R_{1}}^{\sin k},y_{R_{1}}^{\sin k})$, which is fed the exact location using GPS or any other means of location source, and the initial location of Radio2 of sink node ($R_{2}^{\sin k}$) is measured using simple trigonometry, as given in Equation (9). Further Equations (5)–(8) are modified into Equations (10)–(13) to accommodate any initial position and angle of axis of sink node. The initial position of a sink node can help to locate other nodes with their real positions in the coordinate system. Adding the actual location of a sink node solves the first drawback of the DWRL algorithm.

(9) $${(x}_{R_{2}}^{\sin k}{,y}_{R_{2}}^{\sin k}{)\;=\;(x}_{R_{1}}^{\sin k}{+d}_{1}{\cos\theta }_{x}{,y}_{R_{1}}^{\sin k}{+d}_{1}{\sin\theta }_{x})$$

(10a) $$x_{R_{1}}^{n_{2}}{\;=\;r}_{1}{\cos(\theta }_{2}{+\theta }_{x}{)+x}_{R_{1}}^{n_{1}}$$

(10b) $$x_{R_{1}}^{n_{2}^{\prime }}{\;=\;r}_{1}\cos(-\theta_{2}{+\theta }_{x}{)+x}_{R_{1}}^{n_{1}}$$

(11a) $$y_{R_{1}}^{n_{2}}{\;=\;r}_{1}{\sin(\theta }_{2}{+\theta }_{x}{)+y}_{R_{1}}^{n_{1}}$$

(11b) $$y_{R_{1}}^{n_{2}^{\prime }}{\;=\;r}_{1}\sin(-\theta_{2}{+\theta }_{x}{)+y}_{R_{1}}^{n_{1}}$$

(12a) $$x_{R_{2}}^{n_{2}}{\;=\;r}_{3}{\cos(\theta }_{1}{+\theta }_{x}{)+x}_{R_{1}}^{n_{1}}$$

(12b) $$x_{R_{2}}^{n_{2}^{\prime }}{\;=\;r}_{3}\cos(-\theta_{1}{+\theta }_{x}{)+x}_{R_{1}}^{n_{1}}$$

(13a) $$y_{R_{2}}^{n_{2}}{\;=\;r}_{3}{\sin(\theta }_{1}{+\theta }_{x}{)+y}_{R_{1}}^{n_{1}}$$

(13b) $$y_{R_{2}}^{n_{2}^{\prime }}{\;=\;r}_{3}\sin(-\theta_{1}{+\theta }_{x}{)+y}_{R_{1}}^{n_{1}}$$

**Figure 7:**
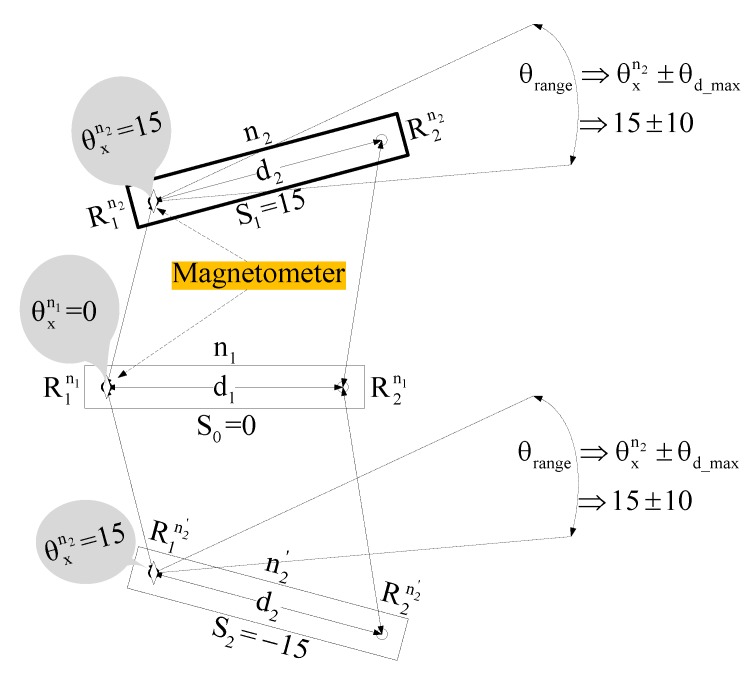
The magnetometer sensor helps to distinguish between actual and another possible location of node $n_{2}$.

A magnetometer is cheap but susceptible to environmental noise, and it may require calibration from time to time. To deal with the problem of magnetometer calibration, we consider that all magnetometers are calibrated well before the localization process starts, and magnetometers should be calibrated whenever required and feasible. To deal with the problem of angle errors due to environmental noise, we consider that we have knowledge of environmental noise and we know the effect of such noise on magnetometer angle deflection. With this knowledge, it appears that we can calculate, in advance, the maximum possible magnetometer angle errors due to environmental noise. If $\theta_{x}^{n_{2}}$ is an angle obtained from magnetometer at $R_{1}^{n_{2}}$ and $\pm \theta_{d\text{\_}\max}$ is the maximum possible angle deflection caused due to environmental noise, then we compare which of the slope of two possible positions of unlocalized node $n_{2}$ falls within angle range $\theta_{\mathrm{range}}={\;\theta }_{x}^{n_{2}}\pm \theta_{d\text{\_}\max}$ is considered as an actual location of $n_{2}$ without performing rigid localization. Figure 7 depicts such a scenario, where slope $S_{1}$ and $S_{2}$ can be found using Equation (14).

(14a) $$S_{1}{=\;\tan}^{-1}(\frac{y_{R_{2}}^{n_{2}}-y_{R_{1}}^{n_{2}}}{x_{R_{2}}^{n_{2}}-x_{R_{1}}^{n_{2}}})$$

(14b) $$S_{2}{=\;\tan}^{-1}(\frac{\left(-y_{R_{2}}^{n_{2}})-(-{\;y}_{R_{1}}^{n_{2}}\right)}{x_{R_{2}}^{n_{2}}-x_{R_{1}}^{n_{2}}})$$

For such semi-localization to produce accurate results of rigid localization without performing rigid localization, the minimum difference between the angle of slope of a localized node and the angle of the magnetometer of an unlocalized node must be greater than $\theta_{d\text{\_}\max}$. As with the use of a magnetometer, we can use only one semi-localization to rigid localize an unlocalized node, therefore, it solves the second and third drawbacks of the DWRL algorithm.

**Figure 8:**
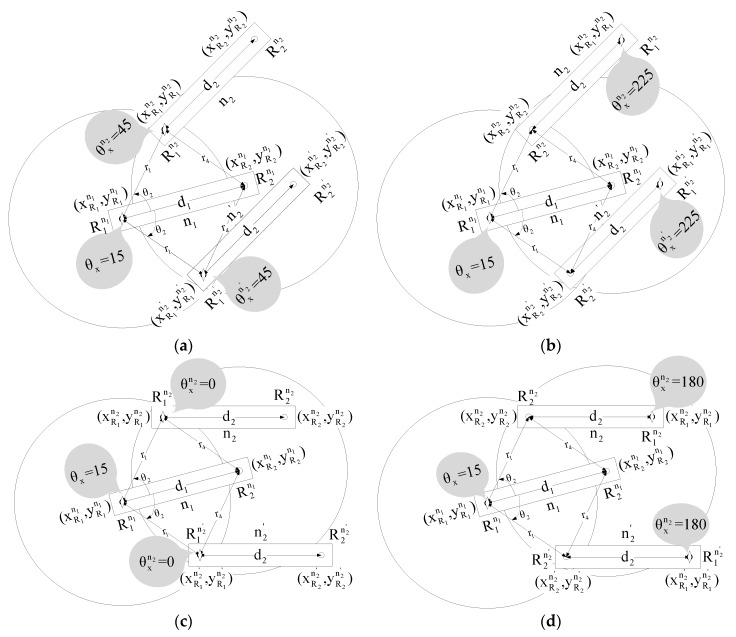
Various cases of connectivity problem. (**a**) Case a. Two radios of a localized node can reach only Radio1 of unlocalized node. (**b**) Case b. Two radios of a localized node can reach only Radio2 of an unlocalized node. (**c**) Case c. Radio1 of a localized node can reach only Radio1 of an unlocalized node, whereas Radio2 of a localized node can reach both radios of an unlocalized node. (**d**) Case d. Radio1 of a localized node can only reach Radio2 of an unlocalized node, whereas Radio2 of a localized node can reach both radios of an unlocalized node.

To overcome the connectivity problem of the DWRL algorithm, we have considered six cases, four of which are shown in Figure 8.

For Case a, the actual and other possible position of $R_{1}^{n_{2}}$ of an unlocalized node can be found with the help of a localized node $n_{1}$ using Equations (4), (10) and (11), whereas the actual and other possible location of $R_{2}^{n_{2}}$ of an unlocalized $n_{2}$ can be found using angle $\theta_{x}^{n_{2}}$ in Equations (15) and (16).

(15a) $$x_{R_{2}}^{n_{2}}{=\;x}_{R_{1}}^{n_{2}}{+d}_{2}{\cos\theta }_{x}^{n_{2}}$$

(15b) $$x_{R_{2}}^{n_{{}_{2}}^{\prime }}{=\;x}_{R_{1}}^{n_{{}_{2}}^{\prime }}{+d}_{2}{\cos\theta }_{x}^{n_{2}}$$

(16a) $$y_{R_{2}}^{n_{2}}{=\;y}_{R_{1}}^{n_{2}}{+d}_{2}{\sin\theta }_{x}^{n_{2}}$$

(16b) $$y_{R_{2}}^{n_{{}_{2}}^{\prime }}{=\;y}_{R_{1}}^{n_{{}_{2}}^{\prime }}{+d}_{2}{\sin\theta }_{x}^{n_{2}}$$

The decision regarding the choosing of the actual location of $n_{2}$ out of two obtained locations is made on the basis that Radio1 and Radio2 of node $n_{1}$ cannot reach Radio2 of node $n_{2}$, but in obtained location $n_{{}_{2}}^{\prime }$ Radio2 of node $n_{{}_{2}}^{\prime }$ lies within the communication range of Radio2 of node $n_{1}$. Mathematically, the actual location of node $n_{2}$ can be confirmed using Equation (17). In Equation (17), if the condition is true then $n_{2}$ is the actual location else $n_{{}_{2}}^{\prime }$.

(17) $$\bar{R_{1}^{n_{1}}R_{2}^{n_{2}}}+\bar{R_{2}^{n_{1}}R_{2}^{n_{2}}}\;>\bar{R_{1}^{n_{1}}R_{2}^{n_{2}^{\prime }}}+\bar{R_{2}^{n_{1}}R_{2}^{n_{2}^{\prime }}}$$

For Case b, the actual and other possible location of $R_{1}^{n_{2}}$ and $R_{2}^{n_{2}}$ of node $n_{2}$ can be found using Equations (4) and (18)–(21). Moreover, we can decide actual position of node $n_{2}$ using Equation (22). In Equation (22) if the condition is true then $n_{2}$ is the actual location else $n_{{}_{2}}^{\prime }$.

(18a) $$x_{R_{2}}^{n_{2}}{=\;r}_{1}{\cos(\theta }_{2}{+\theta }_{x}{)+x}_{R_{1}}^{n_{1}}$$

(18b) $$x_{R_{2}}^{n_{2}^{\prime }}{=\;r}_{1}\cos(-\theta_{2}{+\theta }_{x}{)+x}_{R_{1}}^{n_{1}}$$

(19a) $$y_{R_{2}}^{n_{2}}{=\;r}_{1}{\sin(\theta }_{2}{+\theta }_{x}{)+y}_{R_{1}}^{n_{1}}$$

(19b) $$y_{R_{2}}^{n_{2}^{\prime }}{=\;r}_{1}\sin(-\theta_{2}{+\theta }_{x}{)+y}_{R_{1}}^{n_{1}}$$

(20a) $$x_{R_{1}}^{n_{2}}{=\;x}_{R_{2}}^{n_{2}}{+d}_{2}{\cos(\theta }_{x}^{n_{2}}-180)$$

(20b) $$x_{R_{1}}^{n_{{}_{2}}^{\prime }}{=\;x}_{R_{2}}^{n_{{}_{2}}^{\prime }}{+d}_{2}{\cos(\theta }_{x}^{n_{2}}-180)$$

(21a) $$y_{R_{1}}^{n_{2}}{=\;y}_{R_{2}}^{n_{2}}{+d}_{2}{\sin(\theta }_{x}^{n_{2}}-180)$$

(21b) $$y_{R_{1}}^{n_{{}_{2}}^{\prime }}{=\;y}_{R_{2}}^{n_{{}_{2}}^{\prime }}{+d}_{2}{\sin(\theta }_{x}^{n_{2}}-180)$$

(22) $$\bar{R_{1}^{n_{1}}R_{1}^{n_{2}}}+\bar{R_{2}^{n_{1}}R_{1}^{n_{2}}}\;>\bar{{\;R}_{1}^{n_{1}}R_{1}^{n_{2}^{\prime }}}+\bar{R_{2}^{n_{1}}R_{1}^{n_{2}^{\prime }}}$$

For Case c, the actual and another possible location of node $n_{2}$ is found using Equations (4), (10), (11), (15) and (16), where Equation (23) helps to decide actual position of node $n_{2}$. In Equation (23) if the condition is true then $n_{2}$ is the actual location, otherwise $n_{{}_{2}}^{\prime }$.

(23) $$\bar{R_{1}^{n_{1}}R_{2}^{n_{2}}}+\bar{R_{2}^{n_{1}}R_{2}^{n_{2}}}\;<\bar{{\;R}_{1}^{n_{1}}R_{2}^{n_{2}^{\prime }}}+\bar{R_{2}^{n_{1}}R_{2}^{n_{2}^{\prime }}}$$

For Case d, Equations (4), and (18)–(21) are used to find the actual and other possible locations, where Equation (24) is used to decide actual position of node $n_{2}$. In Equation (24), if the condition is true then $n_{2}$ is the actual location else $n_{{}_{2}}^{\prime }$.

(24) $$\bar{R_{1}^{n_{1}}R_{1}^{n_{2}}}+\bar{R_{2}^{n_{1}}R_{1}^{n_{2}}}\;<\bar{R_{1}^{n_{1}}R_{1}^{n_{2}^{\prime }}}+\bar{R_{2}^{n_{1}}R_{1}^{n_{2}^{\prime }}}$$

Case e and f can also be considered with little variation to Case c and d, where Radio1 of a localized node can reach both radios of an unlocalized node, and Radio2 of a localized node can reach either Radio1 or Radio2 of unlocalized node, respectively. Same equations will be used for Case e and f, as in Case c and d.

All six of these cases are used to solve the connectivity issue of DWRL algorithms in situations where node density or transmission range is not enough.

**Figure 9:**
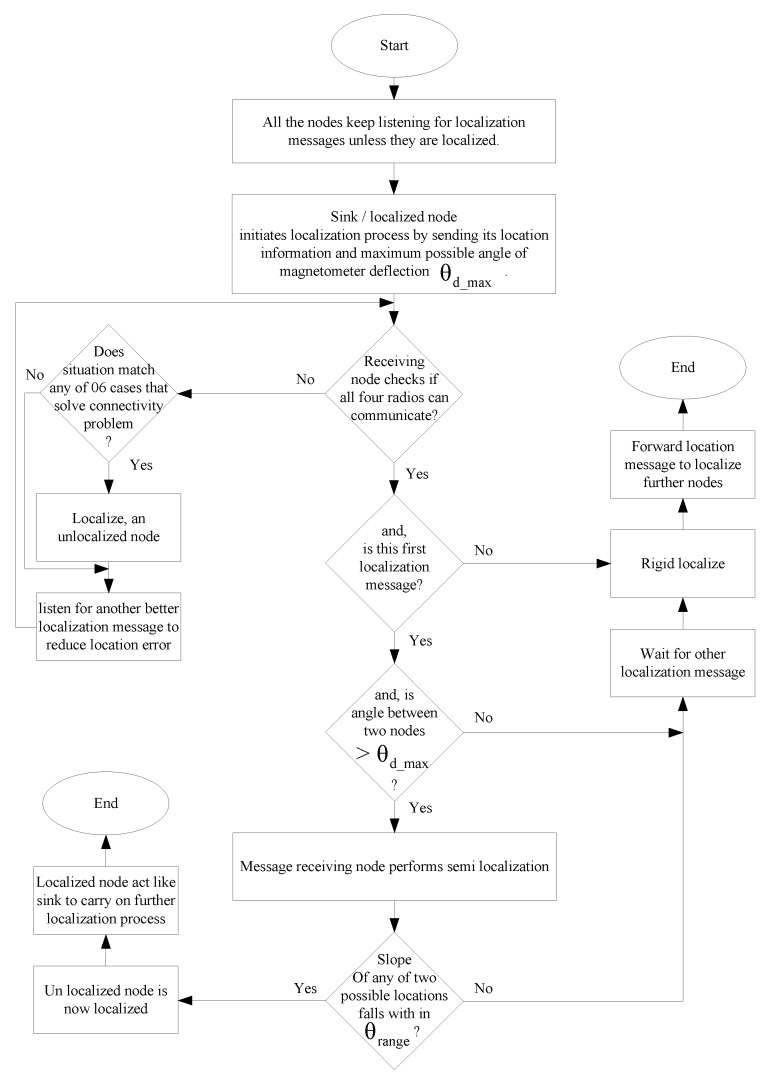
Flow chart of the improved (I)-DWRL algorithm.

A detailed flowchart of the DWRL algorithm is shown in Figure 9.

## 4. Simulation Scenario, Parameters and Results

In this section, we simulate the DWRL and I-DWRL algorithms and analyze the effects of parameters such as range measurement errors, magnetometer errors, transmission range, and so on, where NS-2 [26] is used as a simulation tool. We compare DWRL and I-DWRL in terms of the number of semi-localizations required, the percentage of network nodes localized, and the time and energy required to finish localization process. To accommodate range measurement errors, real-world noise is considered as the summation of high probability small noise and low probability large noise [27,28]. Small noise is modeled as a Gaussian random process as a function of “R”, which is the wireless range of nodes, which is given in Equation (25).

(25) $$N_{S}\left(R\right)\;=\;0.022\;\ln\left(1+R\right)-0.038$$

The low probability large noise value is selected as a function of “R” with probability less than 0.025, which is given in Equation (26).

(26) $$N_{L}\left(R\right)\;=\;0.025\times R$$

Further magnetometer maximum possible deflection from its accurate angle is a joint effect of both noises, and it is modeled as a function of both noises $\theta_{d\text{\_}\max}{(N}_{S}{,N}_{L})$. The effect of both noises is considered in such a way that $0<\theta_{d\text{\_}\max}\leq 45$.

**Figure 10:**
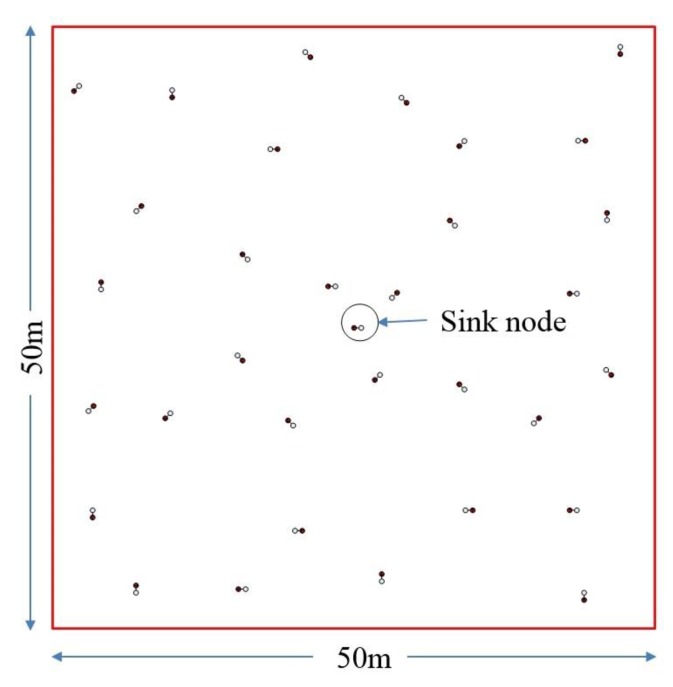
Network simulation scenario.

Figure 10 and Table 1 shows simulation scenario and parameters, respectively.

**Figure 11:**
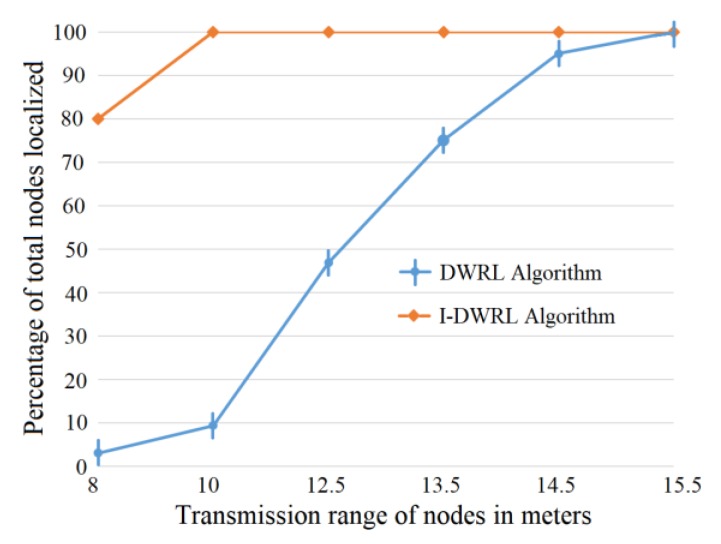
Transmission range versus total percentage of localized nodes.

Figure 11 shows that with lower transmission range connectivity problems can occur, and a smaller number of nodes will be localized with the DWRL algorithm, whereas the I-DWRL algorithm performs well even with the smaller transmission range of nodes. With the transmission range of 8 m, the DWRL algorithm cannot even localize more than 5% of network nodes, whereas our I-DWRL can localize around 80% of network nodes.

**Figure 12:**
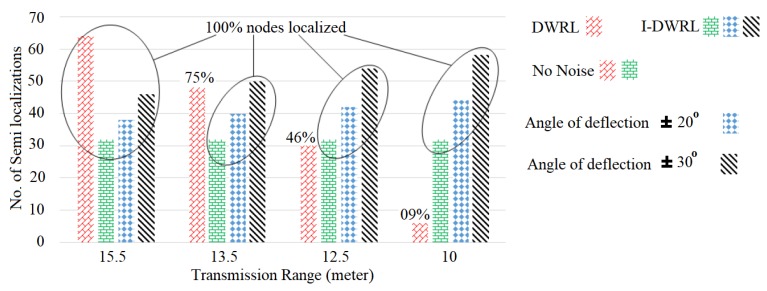
Transmission range versus number of semi-localizations in noiseless and noisy environment.

As we have improved the DWRL algorithm in terms of having a smaller number of semi-localizations required for the full localization of unlocalized nodes, therefore, Figure 12 shows that the I-DWRL algorithm can localize higher percentage of nodes with a lower number of semi-localizations, even in noisy environments. In an ideal case where the transmission range is 15 m and there is no noise, the I-DWRL algorithm can save around 50% of semi-localizations. In the worst case, where the transmission range is 10 m, the DWRL algorithm can reach only 9% of network nodes; therefore, it performs a smaller number of semi-localizations, while I-DWRL can reach 100% of network nodes and performs a greater number of semi-localizations. If the angle of deflection of the magnetometer is increased, then the performance of the I-DWRL algorithm degrades and requires a greater number of semi-localizations.

**Figure 13:**
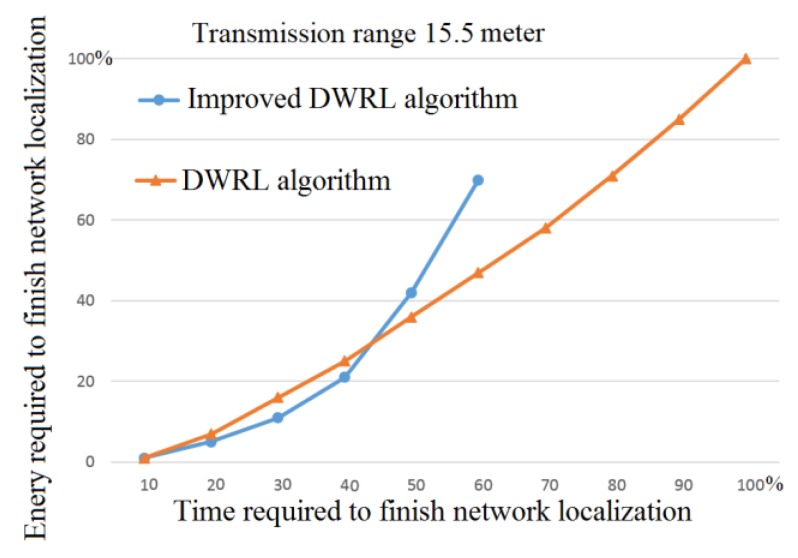
Time and energy graph for full network localization.

For a transmission range of 15.5 m, Figure 13 shows that the I-DWRL algorithm requires less than 60% of network localization time and takes around 70% of the energy to fully localize the same network as with the DWRL algorithm.

**Figure 14:**
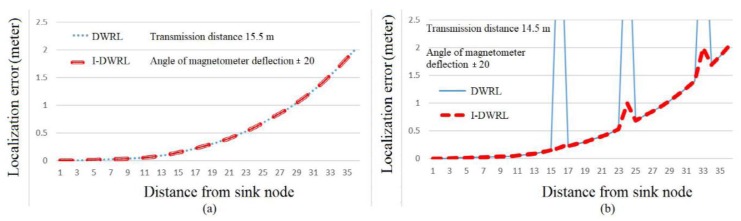
Localization error. (**a**) Without connectivity problem, (**b**) With some connectivity problem.

For the simulation results of localization error, we have considered two transmission ranges, namely, 15.5 m and 14.5 m. The angle of magnetometer deflection due to environmental noise is considered as ±20 degrees. Figure 14a shows that as the distance between sensor nodes and sink nodes is increasing due to multi-hop communication and so is the error, but both the algorithms have same the performance. Figure 14b shows that, due to limited wireless range, the DWRL algorithm cannot reach all network nodes and, for the considered parameters, three nodes cannot be localized. So, for these three nodes the localization error is infinite. Figure 14b shows that the I-DWRL algorithms can reach these three nodes and localize them well.

## 5. Conclusions

The DWRL algorithm uses two UWB radios with each node and requires a minimum of two localized nodes for the localization of an unlocalized node, where all four radios of two communicating nodes should be able to communicate with the remaining three radios. We have improved the DWRL algorithm by using a magnetometer on one of the radios of each node, such that the I-DWRL algorithm requires only one node to fully localize an unlocalized nodes without the strict requirement of connectivity among all four radios of the two communicating nodes. Our results also verify the fact that I-DWRL outperforms in terms of time, energy, number of localized nodes, localization error, and so on. In comparison with the DWRL algorithm, I-DWRL requires almost half the number of semi-localizations in a low-noise environment, 60% of whole network localization time, 70% of the energy, and can localize more number of network nodes with a maintained localization error in cases when DWRL algorithm cannot reach all network nodes.

## Figures and Tables

**Table 1:** Network simulation parameters. Ultra-wide band (UWB).

Simulations Parameter	Value
Area	50 × 50 m^2^
Unlocalized nodes	32
Localized nodes/sink	1
Node distribution	Uniform random distribution
Angle of axis of nodes	random
Different transmission ranges	8, 10, 12.5, 13.5, 14.5, 15.5 m
Inter-radio distance	60 cm
Radio	UWB
UWB Range error without environmental noise	1% of transmission range
